# 1-Ethyl-6-fluoro-7-(4-methyl­piperazin-4-ium-1-yl)-4-oxo-1,4-dihydroquinoline-3-carboxylate hexa­hydrate

**DOI:** 10.1107/S1600536808000421

**Published:** 2008-01-16

**Authors:** Zhe An, Qing-Cheng Liang

**Affiliations:** a School of Pharmaceutical Science, Harbin Medical University, Harbin, 150086, People’s Republic of China; b Second Hospital, Harbin Medical University, Harbin, 150086, People’s Republic of China

## Abstract

In the title compound, C_17_H_20_FN_3_O_3_·6H_2_O, the pefloxacin (pef) neutral zwitterion is accompanied by six water mol­ecules of hydration. An extensive network of O—H⋯O and N—H⋯O hydrogen bonds help to establish the crystal packing.

## Related literature

For metal complexes of the pef anion, see: Baenziger *et al.* (1986 ▶); An, Huang & Qi (2007 ▶); An, Qi & Huang (2007 ▶). For background on the medicinal uses of Hpef, see: Mizuki *et al.* (1996 ▶).
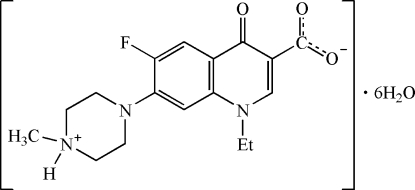


## Experimental

### 

#### Crystal data

C_17_H_20_FN_3_O_3_·6H_2_O
*M* *_r_* = 441.46
Monoclinic, 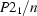

*a* = 8.0925 (15) Å
*b* = 24.075 (5) Å
*c* = 10.8006 (19) Å
β = 92.064 (3)°
*V* = 2102.9 (7) Å^3^
*Z* = 4
Mo *K*α radiation
μ = 0.12 mm^−1^
*T* = 296 (2) K
0.34 × 0.26 × 0.18 mm

#### Data collection

Bruker SMART CCD diffractometer
Absorption correction: multi-scan (*SADABS*; Bruker, 1998 ▶) *T*_min_ = 0.960, *T*_max_ = 0.978
10920 measured reflections
3743 independent reflections
2239 reflections with *I* > 2σ(*I*)
*R*_int_ = 0.051

#### Refinement

*R*[*F* ^2^ > 2σ(*F* ^2^)] = 0.048
*wR*(*F* ^2^) = 0.134
*S* = 1.02
3743 reflections
312 parameters
19 restraints
H atoms treated by a mixture of independent and constrained refinement
Δρ_max_ = 0.22 e Å^−3^
Δρ_min_ = −0.20 e Å^−3^

### 

Data collection: *SMART* (Bruker, 1998 ▶); cell refinement: *SAINT-Plus* (Bruker, 1998 ▶); data reduction: *SAINT-Plus*; program(s) used to solve structure: *SHELXS97* (Sheldrick, 2008 ▶); program(s) used to refine structure: *SHELXL97* (Sheldrick, 2008 ▶); molecular graphics: *SHELXTL* (Bruker, 1998 ▶); software used to prepare material for publication: *SHELXTL*.

## Supplementary Material

Crystal structure: contains datablocks I, global. DOI: 10.1107/S1600536808000421/hb2680sup1.cif

Structure factors: contains datablocks I. DOI: 10.1107/S1600536808000421/hb2680Isup2.hkl

Additional supplementary materials:  crystallographic information; 3D view; checkCIF report

## Figures and Tables

**Table 1:** Hydrogen-bond geometry (Å, °)

*D*—H⋯*A*	*D*—H	H⋯*A*	*D*⋯*A*	*D*—H⋯*A*
O1*W*—H1*W*1⋯O1	0.857 (10)	1.866 (12)	2.700 (3)	164 (3)
O1*W*—H1*W*2⋯O3*W*	0.85 (3)	2.201 (16)	3.017 (3)	162 (3)
O2*W*—H2*W*1⋯O2	0.851 (10)	1.881 (13)	2.722 (3)	170 (4)
O2*W*—H2*W*2⋯O6*W*	0.86 (3)	1.91 (3)	2.765 (3)	171 (3)
O3*W*—H3*W*2⋯O1^i^	0.852 (10)	1.896 (13)	2.730 (3)	166 (3)
O3*W*—H3*W*1⋯O2*W*	0.86 (3)	1.82 (3)	2.679 (3)	175 (3)
O6*W*—H6*W*1⋯O1*W*^i^	0.86 (3)	1.92 (3)	2.765 (4)	170 (4)
O6*W*—H6*W*2⋯O4*W*^ii^	0.847 (10)	2.17 (3)	3.007 (4)	170 (4)
N3—H3N⋯O3*W*^iii^	0.910 (10)	1.847 (13)	2.730 (3)	163 (3)
O4*W*—H4*W*1⋯O3	0.86 (3)	1.89 (3)	2.739 (3)	169 (3)
O4*W*—H4*W*2⋯O5*W*^ii^	0.85 (3)	1.959 (15)	2.783 (3)	163 (3)
O5*W*—H5*W*1⋯O2	0.85 (3)	1.97 (3)	2.792 (3)	164 (4)
O5*W*—H5*W*1⋯O3	0.85 (3)	2.63 (4)	3.142 (3)	120 (3)
O5*W*—H5*W*2⋯O5*W*^ii^	0.84 (3)	2.06 (2)	2.769 (5)	142 (3)

Symmetry codes: (i) 
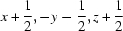
; (ii) 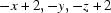; (iii) 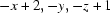.

**Fig. 1.**
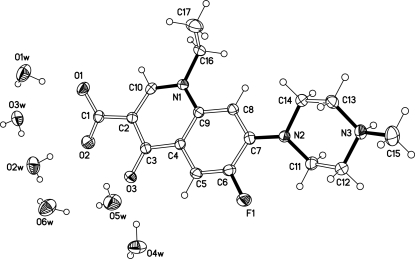
The asymmetric unit of (I), showing 50% displacement ellipsoids.

## supplementary crystallographic information

### Comment

Pefloxacin (Hpef, C_17_H_20_FN_3_O_3_, 1-ethyl-6-fluoro-7-(4-methylpiperazin-1-yl)-4-oxo-quinoline -3-carboxylic acid) is member of a class of quinolones used to treat infections (Mizuki *et al.*, 1996). The silver(I), manganese(II) and cobalt(II) derivatives of the pefloxacin (pef) anion have been reported (Baenziger *et al.*, 1986; An, Huang & Qi, 2007; An, Qi & Huang, 2007).

We attempted to prepare a nickel(II) complex of pef, but the title compound, (I), arose instead. The neutral Hpef zwitterion shows nominal proton transfer from O1 or O2 to N3. Consequently the C1—O1 [1.264 (3) Å] and C1—O2 [1.245 (3) Å] bond lengths are very similar. The bond angle sum for N1 of 360° indicates *sp*^2^ hybridization for this atom. The N2/N3/C11—C14 ring is a typical chair.

The components of (I) are linked by O—H···O and O—H···N hydrogen bonds (Table 1) involving all the potential donors, generating a three-dimensional supramolecular network.

### Experimental

A mixture of Ni(NO_3_)_2_.6H_2_O (0.075 g, 0.25 mmol), Hpef (0.17 g, 0.5 mmol), and water (12 ml) was stirred for 30 min in air. The mixture was then transferred to a 23 ml Teflon-lined hydrothermal bomb. The bomb was kept at 423 K for 72 h under autogenous pressure. The targeted Ni^2+^ complex was not synthesized and colorless prisms of (I) were obtained from the reaction mixture after cooling.

### Refinement

The carbon-bound H atoms were positioned geometrically (C—H = 0.93–0.97 Å) and were included in the refinement in the riding model approximation, with *U*_iso_(H) = 1.2*U*_eq_(C). The O– and N-bonded H atoms were located in a difference map, and were refined with a distance restraint of N—H = 0.90 (1) /%A and with *U*_iso_(H) = 1.5*U*_eq_(N) and O—H = 0.85 (1) /%A and with *U*_iso_(H) = 1.5*U*_eq_(O). Some short intermolecular H···H contacts occur; thus, the H atom positions of the water molecules should be regarded as less reliable.

### Crystal data

**Table:**

C_17_H_20_FN_3_O_3_·6H_2_O	*F*_000_ = 944
*M**_r_* = 441.46	*D*_x_ = 1.394 Mg m^−^^3^
Monoclinic, *P*2_1_/*n*	Mo *K*α radiation λ = 0.71073 Å
Hall symbol: -P 2yn	Cell parameters from 10069 reflections
*a* = 8.0925 (15) Å	θ = 2.1–25.1º
*b* = 24.075 (5) Å	µ = 0.12 mm^−^^1^
*c* = 10.8006 (19) Å	*T* = 296 (2) K
β = 92.064 (3)º	Prism, colorless
*V* = 2102.9 (7) Å^3^	0.34 × 0.26 × 0.18 mm
*Z* = 4	

### Data collection

**Table:**

Bruker SMART CCD diffractometer	3743 independent reflections
Radiation source: fine-focus sealed tube	2239 reflections with *I* > 2σ(*I*)
Monochromator: graphite	*R*_int_ = 0.051
*T* = 295(2) K	θ_max_ = 25.1º
ω scans	θ_min_ = 2.1º
Absorption correction: multi-scan(SADABS; Bruker, 1998)	*h* = −9→9
*T*_min_ = 0.960, *T*_max_ = 0.978	*k* = −25→28
10920 measured reflections	*l* = −8→12

### Refinement

**Table:**

Refinement on *F*^2^	Secondary atom site location: difference Fourier map
Least-squares matrix: full	Hydrogen site location: inferred from neighbouring sites
*R*[*F*^2^ > 2σ(*F*^2^)] = 0.048	H atoms treated by a mixture of independent and constrained refinement
*wR*(*F*^2^) = 0.134	*w* = 1/[σ^2^(*F*_o_^2^) + (0.0593*P*)^2^ + 0.0315*P*] where *P* = (*F*_o_^2^ + 2*F*_c_^2^)/3
*S* = 1.02	(Δ/σ)_max_ < 0.001
3743 reflections	Δρ_max_ = 0.22 e Å^−^^3^
312 parameters	Δρ_min_ = −0.20 e Å^−^^3^
19 restraints	Extinction correction: none
Primary atom site location: structure-invariant direct methods	

### Special details

**Table:**

Geometry. All e.s.d.'s (except the e.s.d. in the dihedral angle between two l.s. planes) are estimated using the full covariance matrix. The cell e.s.d.'s are taken into account individually in the estimation of e.s.d.'s in distances, angles and torsion angles; correlations between e.s.d.'s in cell parameters are only used when they are defined by crystal symmetry. An approximate (isotropic) treatment of cell e.s.d.'s is used for estimating e.s.d.'s involving l.s. planes.

### Fractional atomic coordinates and isotropic or equivalent isotropic displacement parameters (Å^2^)

**Table:**

	*x*	*y*	*z*	*U*_iso_*/*U*_eq_	
F1	0.4982 (2)	0.12293 (6)	0.56081 (13)	0.0524 (5)	
O1	0.9475 (3)	−0.18675 (8)	0.62111 (17)	0.0512 (5)	
O2	0.9816 (3)	−0.13364 (8)	0.78658 (17)	0.0548 (6)	
O3	0.7598 (2)	−0.04158 (7)	0.76314 (16)	0.0436 (5)	
O1W	1.0319 (4)	−0.28815 (9)	0.7094 (2)	0.0761 (7)	
H1W1	1.010 (5)	−0.2538 (6)	0.695 (3)	0.114*	
H1W2	1.104 (4)	−0.2906 (13)	0.768 (3)	0.114*	
O2W	1.0567 (3)	−0.19330 (10)	0.9952 (2)	0.0687 (7)	
H2W1	1.042 (4)	−0.1772 (13)	0.9256 (17)	0.103*	
H2W2	1.121 (4)	−0.1734 (12)	1.042 (2)	0.103*	
O3W	1.2764 (3)	−0.26876 (9)	0.92250 (17)	0.0558 (6)	
H3W2	1.318 (4)	−0.2872 (11)	0.9835 (19)	0.084*	
H3W1	1.204 (3)	−0.2460 (12)	0.950 (2)	0.084*	
O4W	0.6261 (3)	0.02774 (10)	0.9339 (2)	0.0637 (6)	
O5W	1.0677 (3)	−0.04707 (12)	0.9484 (2)	0.0817 (8)	
H5W1	1.022 (4)	−0.0705 (13)	0.899 (3)	0.123*	
H5W2	0.997 (3)	−0.0308 (15)	0.990 (4)	0.123*	
O6W	1.2719 (3)	−0.13973 (11)	1.1601 (3)	0.0914 (9)	
H6W1	1.360 (3)	−0.1585 (13)	1.176 (4)	0.137*	
H6W2	1.298 (5)	−0.1066 (7)	1.143 (4)	0.137*	
N1	0.8676 (2)	−0.05154 (8)	0.39571 (18)	0.0325 (5)	
N2	0.5711 (3)	0.12232 (8)	0.31502 (18)	0.0351 (5)	
N3	0.4587 (3)	0.20866 (9)	0.1516 (2)	0.0399 (6)	
H3N	0.552 (2)	0.2224 (11)	0.119 (2)	0.060*	
C1	0.9405 (3)	−0.14034 (11)	0.6754 (3)	0.0361 (6)	
C2	0.8807 (3)	−0.09193 (10)	0.6006 (2)	0.0318 (6)	
C3	0.7935 (3)	−0.04596 (10)	0.6512 (2)	0.0320 (6)	
C4	0.7415 (3)	−0.00337 (10)	0.5619 (2)	0.0303 (6)	
C5	0.6494 (3)	0.04228 (10)	0.6011 (2)	0.0350 (6)	
H5	0.6234	0.0454	0.6840	0.042*	
C6	0.5981 (3)	0.08168 (11)	0.5202 (2)	0.0354 (6)	
C7	0.6356 (3)	0.08101 (11)	0.3938 (2)	0.0327 (6)	
C8	0.7274 (3)	0.03613 (10)	0.3540 (2)	0.0318 (6)	
H8	0.7556	0.0341	0.2714	0.038*	
C9	0.7785 (3)	−0.00614 (10)	0.4361 (2)	0.0294 (6)	
C10	0.9112 (3)	−0.09195 (10)	0.4773 (2)	0.0337 (6)	
H10	0.9671	−0.1225	0.4467	0.040*	
C11	0.6038 (3)	0.18058 (10)	0.3458 (2)	0.0404 (7)	
H11B	0.7100	0.1916	0.3149	0.048*	
H11A	0.6076	0.1853	0.4351	0.048*	
C12	0.4696 (4)	0.21632 (11)	0.2885 (2)	0.0422 (7)	
H12B	0.3646	0.2067	0.3233	0.051*	
H12A	0.4924	0.2550	0.3076	0.051*	
C13	0.4413 (3)	0.14871 (11)	0.1185 (2)	0.0390 (7)	
H13B	0.4475	0.1445	0.0295	0.047*	
H13A	0.3338	0.1355	0.1424	0.047*	
C14	0.5745 (3)	0.11382 (11)	0.1818 (2)	0.0391 (7)	
H14B	0.5560	0.0749	0.1627	0.047*	
H14A	0.6818	0.1243	0.1522	0.047*	
C15	0.3210 (4)	0.24236 (14)	0.0946 (3)	0.0607 (9)	
H15A	0.3180	0.2374	0.0064	0.091*	
H15B	0.3387	0.2809	0.1138	0.091*	
H15C	0.2179	0.2305	0.1270	0.091*	
C16	0.9107 (3)	−0.05917 (12)	0.2649 (2)	0.0390 (7)	
H16B	1.0083	−0.0824	0.2617	0.047*	
H16A	0.9375	−0.0233	0.2299	0.047*	
C17	0.7735 (4)	−0.08525 (15)	0.1874 (3)	0.0642 (10)	
H17A	0.7489	−0.1213	0.2198	0.096*	
H17B	0.8072	−0.0888	0.1035	0.096*	
H17C	0.6766	−0.0623	0.1895	0.096*	
H4W1	0.657 (4)	0.0071 (13)	0.874 (2)	0.096*	
H4W2	0.709 (3)	0.0325 (14)	0.984 (2)	0.096*	

### Atomic displacement parameters (Å^2^)

**Table:**

	*U*^11^	*U*^22^	*U*^33^	*U*^12^	*U*^13^	*U*^23^
F1	0.0657 (11)	0.0498 (11)	0.0420 (10)	0.0227 (9)	0.0072 (8)	−0.0003 (7)
O1	0.0692 (14)	0.0320 (12)	0.0515 (13)	0.0057 (10)	−0.0116 (10)	0.0010 (9)
O2	0.0748 (15)	0.0505 (13)	0.0380 (13)	0.0082 (11)	−0.0155 (10)	0.0037 (9)
O3	0.0604 (13)	0.0411 (12)	0.0293 (11)	0.0039 (10)	0.0017 (9)	0.0017 (8)
O1W	0.097 (2)	0.0451 (14)	0.0843 (19)	−0.0009 (14)	−0.0253 (14)	0.0077 (12)
O2W	0.0744 (18)	0.0650 (16)	0.0660 (16)	0.0009 (13)	−0.0099 (13)	0.0230 (12)
O3W	0.0567 (15)	0.0574 (15)	0.0531 (14)	−0.0038 (11)	−0.0015 (10)	0.0151 (10)
O4W	0.0667 (15)	0.0719 (17)	0.0519 (15)	0.0112 (13)	−0.0060 (11)	−0.0213 (11)
O5W	0.091 (2)	0.081 (2)	0.0720 (19)	−0.0046 (16)	−0.0108 (14)	−0.0229 (12)
O6W	0.107 (2)	0.0690 (18)	0.096 (2)	0.0147 (16)	−0.0310 (17)	−0.0081 (16)
N1	0.0363 (13)	0.0324 (12)	0.0290 (12)	0.0011 (10)	0.0018 (9)	−0.0018 (10)
N2	0.0484 (14)	0.0282 (12)	0.0280 (12)	−0.0011 (11)	−0.0074 (10)	−0.0007 (9)
N3	0.0408 (15)	0.0389 (14)	0.0400 (14)	0.0036 (11)	0.0015 (11)	0.0076 (10)
C1	0.0346 (16)	0.0341 (16)	0.0395 (17)	−0.0036 (13)	−0.0005 (12)	0.0017 (13)
C2	0.0330 (15)	0.0322 (15)	0.0298 (15)	−0.0020 (12)	−0.0040 (11)	0.0008 (11)
C3	0.0324 (15)	0.0327 (15)	0.0305 (15)	−0.0051 (12)	−0.0031 (11)	−0.0002 (12)
C4	0.0325 (15)	0.0285 (14)	0.0295 (14)	−0.0049 (12)	−0.0025 (11)	−0.0001 (11)
C5	0.0409 (16)	0.0380 (16)	0.0258 (14)	0.0016 (13)	−0.0013 (11)	−0.0029 (12)
C6	0.0377 (16)	0.0359 (16)	0.0325 (15)	0.0056 (13)	−0.0003 (12)	−0.0046 (12)
C7	0.0316 (14)	0.0332 (15)	0.0327 (15)	−0.0062 (12)	−0.0045 (11)	0.0026 (12)
C8	0.0338 (15)	0.0317 (15)	0.0299 (14)	−0.0024 (12)	0.0026 (11)	0.0000 (11)
C9	0.0278 (14)	0.0304 (14)	0.0299 (14)	−0.0053 (12)	0.0009 (11)	−0.0011 (11)
C10	0.0340 (15)	0.0266 (14)	0.0405 (16)	0.0019 (12)	−0.0015 (12)	−0.0020 (12)
C11	0.0492 (18)	0.0333 (16)	0.0378 (16)	−0.0046 (14)	−0.0090 (12)	−0.0014 (12)
C12	0.0522 (19)	0.0367 (16)	0.0379 (17)	0.0030 (14)	0.0035 (13)	−0.0014 (12)
C13	0.0450 (17)	0.0415 (17)	0.0304 (15)	−0.0026 (14)	−0.0021 (12)	0.0020 (12)
C14	0.0504 (17)	0.0338 (16)	0.0329 (16)	0.0014 (13)	−0.0010 (12)	0.0005 (12)
C15	0.053 (2)	0.061 (2)	0.068 (2)	0.0162 (17)	−0.0062 (16)	0.0190 (16)
C16	0.0476 (17)	0.0404 (16)	0.0294 (15)	0.0017 (13)	0.0086 (12)	−0.0007 (12)
C17	0.073 (2)	0.077 (2)	0.0430 (19)	−0.019 (2)	0.0019 (16)	−0.0111 (16)

### Geometric parameters (Å, °)

**Table:**

F1—C6	1.363 (3)	C3—C4	1.459 (3)
C1—O1	1.264 (3)	C4—C5	1.402 (3)
C1—O2	1.245 (3)	C4—C9	1.404 (3)
C3—O3	1.253 (3)	C5—C6	1.345 (3)
O1W—H1W1	0.857 (10)	C5—H5	0.9300
O1W—H1W2	0.85 (3)	C6—C7	1.409 (3)
O2W—H2W1	0.851 (10)	C7—C8	1.388 (3)
O2W—H2W2	0.86 (3)	C8—C9	1.402 (3)
O3W—H3W2	0.852 (10)	C8—H8	0.9300
O3W—H3W1	0.86 (3)	C10—H10	0.9300
O4W—H4W1	0.86 (3)	C11—C12	1.501 (4)
O4W—H4W2	0.851 (10)	C11—H11B	0.9700
O5W—H5W1	0.85 (3)	C11—H11A	0.9700
O5W—H5W2	0.84 (3)	C12—H12B	0.9700
O6W—H6W1	0.86 (3)	C12—H12A	0.9700
O6W—H6W2	0.847 (10)	C13—C14	1.510 (4)
N1—C10	1.351 (3)	C13—H13B	0.9700
N1—C9	1.388 (3)	C13—H13A	0.9700
N1—C16	1.479 (3)	C14—H14B	0.9700
N2—C7	1.397 (3)	C14—H14A	0.9700
N2—C14	1.454 (3)	C15—H15A	0.9600
N2—C11	1.463 (3)	C15—H15B	0.9600
N3—C12	1.490 (3)	C15—H15C	0.9600
N3—C13	1.492 (3)	C16—C17	1.504 (4)
N3—C15	1.493 (3)	C16—H16B	0.9700
N3—H3N	0.910 (10)	C16—H16A	0.9700
C1—C2	1.489 (4)	C17—H17A	0.9600
C2—C10	1.363 (3)	C17—H17B	0.9600
C2—C3	1.431 (3)	C17—H17C	0.9600
			
H1W1—O1W—H1W2	109.3 (17)	N1—C10—C2	125.8 (2)
H2W1—O2W—H2W2	109.4 (16)	N1—C10—H10	117.1
H3W2—O3W—H3W1	108.6 (16)	C2—C10—H10	117.1
H4W1—O4W—H4W2	108.2 (16)	N2—C11—C12	109.5 (2)
H5W1—O5W—H5W2	110.9 (18)	N2—C11—H11B	109.8
H6W1—O6W—H6W2	109.4 (17)	C12—C11—H11B	109.8
C10—N1—C9	119.2 (2)	N2—C11—H11A	109.8
C10—N1—C16	118.0 (2)	C12—C11—H11A	109.8
C9—N1—C16	122.7 (2)	H11B—C11—H11A	108.2
C7—N2—C14	118.8 (2)	N3—C12—C11	110.8 (2)
C7—N2—C11	118.89 (19)	N3—C12—H12B	109.5
C14—N2—C11	110.49 (19)	C11—C12—H12B	109.5
C12—N3—C13	111.04 (19)	N3—C12—H12A	109.5
C12—N3—C15	111.0 (2)	C11—C12—H12A	109.5
C13—N3—C15	111.4 (2)	H12B—C12—H12A	108.1
C12—N3—H3N	108.4 (19)	N3—C13—C14	111.7 (2)
C13—N3—H3N	109.5 (19)	N3—C13—H13B	109.3
C15—N3—H3N	105.3 (19)	C14—C13—H13B	109.3
O2—C1—O1	123.2 (2)	N3—C13—H13A	109.3
O2—C1—C2	119.5 (2)	C14—C13—H13A	109.3
O1—C1—C2	117.3 (2)	H13B—C13—H13A	107.9
C10—C2—C3	118.9 (2)	N2—C14—C13	109.3 (2)
C10—C2—C1	117.6 (2)	N2—C14—H14B	109.8
C3—C2—C1	123.6 (2)	C13—C14—H14B	109.8
O3—C3—C2	124.1 (2)	N2—C14—H14A	109.8
O3—C3—C4	120.7 (2)	C13—C14—H14A	109.8
C2—C3—C4	115.2 (2)	H14B—C14—H14A	108.3
C5—C4—C9	117.6 (2)	N3—C15—H15A	109.5
C5—C4—C3	119.7 (2)	N3—C15—H15B	109.5
C9—C4—C3	122.7 (2)	H15A—C15—H15B	109.5
C6—C5—C4	120.8 (2)	N3—C15—H15C	109.5
C6—C5—H5	119.6	H15A—C15—H15C	109.5
C4—C5—H5	119.6	H15B—C15—H15C	109.5
C5—C6—F1	118.5 (2)	N1—C16—C17	112.9 (2)
C5—C6—C7	123.3 (2)	N1—C16—H16B	109.0
F1—C6—C7	118.1 (2)	C17—C16—H16B	109.0
C8—C7—N2	123.9 (2)	N1—C16—H16A	109.0
C8—C7—C6	116.4 (2)	C17—C16—H16A	109.0
N2—C7—C6	119.5 (2)	H16B—C16—H16A	107.8
C7—C8—C9	121.2 (2)	C16—C17—H17A	109.5
C7—C8—H8	119.4	C16—C17—H17B	109.5
C9—C8—H8	119.4	H17A—C17—H17B	109.5
N1—C9—C8	121.2 (2)	C16—C17—H17C	109.5
N1—C9—C4	118.2 (2)	H17A—C17—H17C	109.5
C8—C9—C4	120.6 (2)	H17B—C17—H17C	109.5
			
O2—C1—C2—C10	149.2 (3)	C10—N1—C9—C8	−178.5 (2)
O1—C1—C2—C10	−30.5 (3)	C16—N1—C9—C8	−1.6 (3)
O2—C1—C2—C3	−30.8 (4)	C10—N1—C9—C4	1.9 (3)
O1—C1—C2—C3	149.5 (2)	C16—N1—C9—C4	178.8 (2)
C10—C2—C3—O3	−179.6 (2)	C7—C8—C9—N1	178.8 (2)
C1—C2—C3—O3	0.5 (4)	C7—C8—C9—C4	−1.7 (3)
C10—C2—C3—C4	1.5 (3)	C5—C4—C9—N1	−179.1 (2)
C1—C2—C3—C4	−178.4 (2)	C3—C4—C9—N1	0.3 (3)
O3—C3—C4—C5	−1.5 (3)	C5—C4—C9—C8	1.3 (3)
C2—C3—C4—C5	177.4 (2)	C3—C4—C9—C8	−179.3 (2)
O3—C3—C4—C9	179.1 (2)	C9—N1—C10—C2	−2.5 (4)
C2—C3—C4—C9	−2.0 (3)	C16—N1—C10—C2	−179.6 (2)
C9—C4—C5—C6	0.3 (4)	C3—C2—C10—N1	0.6 (4)
C3—C4—C5—C6	−179.1 (2)	C1—C2—C10—N1	−179.4 (2)
C4—C5—C6—F1	175.0 (2)	C7—N2—C11—C12	−154.7 (2)
C4—C5—C6—C7	−1.7 (4)	C14—N2—C11—C12	62.7 (3)
C14—N2—C7—C8	11.0 (4)	C13—N3—C12—C11	53.2 (3)
C11—N2—C7—C8	−128.5 (3)	C15—N3—C12—C11	177.7 (2)
C14—N2—C7—C6	−164.6 (2)	N2—C11—C12—N3	−58.1 (3)
C11—N2—C7—C6	56.0 (3)	C12—N3—C13—C14	−52.3 (3)
C5—C6—C7—C8	1.3 (4)	C15—N3—C13—C14	−176.6 (2)
F1—C6—C7—C8	−175.4 (2)	C7—N2—C14—C13	156.2 (2)
C5—C6—C7—N2	177.2 (2)	C11—N2—C14—C13	−61.2 (3)
F1—C6—C7—N2	0.4 (3)	N3—C13—C14—N2	56.0 (3)
N2—C7—C8—C9	−175.3 (2)	C10—N1—C16—C17	93.5 (3)
C6—C7—C8—C9	0.4 (3)	C9—N1—C16—C17	−83.4 (3)

### Hydrogen-bond geometry (Å, °)

**Table:**

*D*—H···*A*	*D*—H	H···*A*	*D*···*A*	*D*—H···*A*
O1W—H1W1···O1	0.857 (10)	1.866 (12)	2.700 (3)	164 (3)
O1W—H1W2···O3W	0.85 (3)	2.201 (16)	3.017 (3)	162 (3)
O2W—H2W1···O2	0.851 (10)	1.881 (13)	2.722 (3)	170 (4)
O2W—H2W2···O6W	0.86 (3)	1.91 (3)	2.765 (3)	171 (3)
O3W—H3W2···O1^i^	0.852 (10)	1.896 (13)	2.730 (3)	166 (3)
O3W—H3W1···O2W	0.86 (3)	1.82 (3)	2.679 (3)	175 (3)
O6W—H6W1···O1W^i^	0.86 (3)	1.92 (3)	2.765 (4)	170 (4)
O6W—H6W2···O4W^ii^	0.847 (10)	2.17 (3)	3.007 (4)	170 (4)
N3—H3N···O3W^iii^	0.910 (10)	1.847 (13)	2.730 (3)	163 (3)
O4W—H4W1···O3	0.86 (3)	1.89 (3)	2.739 (3)	169 (3)
O4W—H4W2···O5W^ii^	0.85 (3)	1.959 (15)	2.783 (3)	163 (3)
O5W—H5W1···O2	0.85 (3)	1.97 (3)	2.792 (3)	164 (4)
O5W—H5W1···O3	0.85 (3)	2.63 (4)	3.142 (3)	120 (3)
O5W—H5W2···O5W^ii^	0.84 (3)	2.06 (2)	2.769 (5)	142 (3)

Symmetry codes: (i) *x*+1/2, −*y*−1/2, *z*+1/2; (ii) −*x*+2, −*y*, −*z*+2; (iii) −*x*+2, −*y*, −*z*+1.
